# Advances in polarised luminescence approaches to understanding interactions between biomolecules

**DOI:** 10.1042/BST20253138

**Published:** 2026-04-02

**Authors:** Edan Habel, Pinky Vishwakarma, Thomas Huber, Alison Rodger

**Affiliations:** Research School of Chemistry, The Australian National University, Canberra, 2601 ACT, Australia

**Keywords:** Biomolecular interactions, Circularly polarised luminescence, Fluorescence detected circular dichroism, Fluorescence detected linear dichroism, fluorescence polarisation anisotropy, Linearly polarised luminescence, Luminescence spectroscopy

## Abstract

Luminescence spectroscopies are attractive due to their sensitivity and selectivity. Polarised light provides added dimensions to luminescence data, leading to techniques that provide information about molecular structure and interactions. In this review the principles of steady-state fluorescence techniques, including fluorescence-detected circular dichroism, fluorescence-detected linear dichroism, fluorescence polarisation anisotropy, circularly polarised luminescence, and linearly polarised luminescence, are outlined and illustrated with examples of how they have been used to study biomolecules and their interactions with other molecules.

## Introduction

Luminescence occurs when an electronic excited state of a molecule loses its energy by emitting a photon. We usually divide luminescence into fluorescence where the light is emitted quickly (within nanoseconds) and phosphorescence where radiation is slower. In solution phase, fluorescence generally occurs after radiationless decay to the ground vibrational state of the first excited state (of the same multiplicity as the ground state), which is referred to as Kasha’s rule [1]. For phosphorescence, the excited molecule transitions to a longer-lived, metastable state (usually of a different spin) before returning to the ground state. Factoring in the polarisation of light enhances the information content when analysing luminescence readouts. This review focuses on what can be achieved for biomolecules by measuring luminescence emitted by a sample while it is continuously illuminated with a constant light source where either the incident or the emitted light is circularly or linearly polarised. Fluorescence-detected circular and linear dichroism (FDCD and FDLD) use polarised incident light and count the emitted photons. Alternatively, circularly and linearly polarised luminescence (CPL or LPL) use unpolarised incident light and detect polarisations of emitted light. Fluorescence polarisation anisotropy (FPA) involves linear polarisation of both the incident light and the emitted light.

Jameson and Ross [2] credited the first observation of FPA to Weigert in 1920 [3]. Longhi et al. [4] thought that CPL was probably first measured in 1948 by Samilov [5]. To this date, the experiments are challenging to undertake, and consequently, the literature on CPL or FPA remains limited and is scattered over decades. For example, wavelength scanning LPL spectroscopy has significant potential, but as far as we are aware, our 2024 paper [6] is the first published work covering it. This review is intended to illustrate key insights and theory in this field, rather than act as a comprehensive review. Data collected on our instruments are included to illustrate some of the points.

## Unpolarised light fluorescence

**Figure 1:**
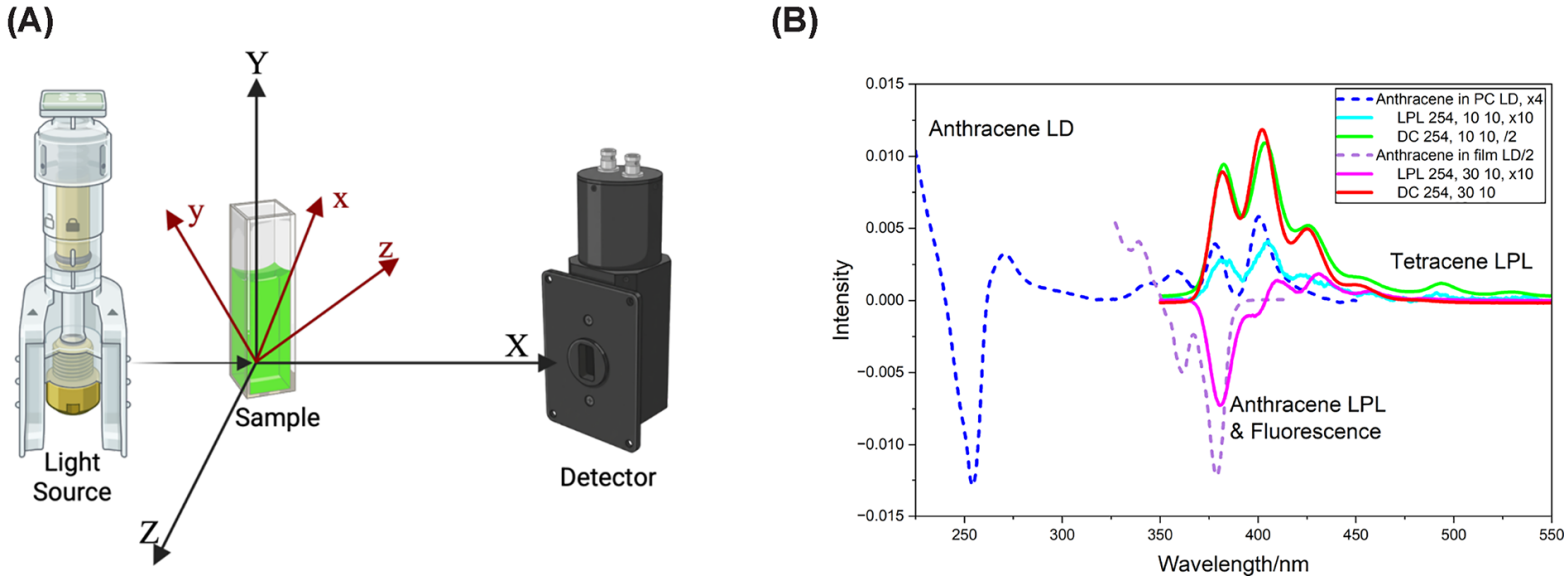
Axis systems used in this work and example LD and LPL spectra. (**A**) Axis systems used in this work except for FPA. {X, Y, Z} is the laboratory-fixed system: X is the direction of light propagation, Y the vertical direction, and Z is the horizontal direction. {x, y, z} is the molecular frame: the z-direction is the principal component of the molecule, while x is the least oriented. (**B**) Anthracene LD, LPL and fluorescence (DC) for anthracene embedded in phosphatidyl choline (PC) flow-oriented liposomes and a stretched polyethylene film. Liposomes are prepared by sonication, which typically produces polydisperse structures with diameters below 100 nm [7,8]. Data are shown for excitation into the 254 nm band. (380 nm data are more intense but the excitation and emission overlap). DC is the fluorescence in volts. Blue/green spectra are for liposomes and red/pink for films.”30/10”, for example, denotes excitation bandwidth of 30 nm and emission bandwidth of 10. “x4”, for example, denotes intensity scaled by a factor of 4 for visualisation purposes. The film LD (dashed purple) 0–0 component at about 378 nm is negative in accord with it being short-axis polarised. It has a vibronic series progressing to shorter wavelengths and changing sign after two components, reflecting the significant coupling between the short-axis polarised 378 nm band and the 254 nm long-axis polarised band. The film LPL (solid pink) begins with a negative 379 nm band, and its vibronic series goes to longer wavelengths, also changing sign after two components. In the Couette-flow liposomes, the anthracene is oriented perpendicular to the flow direction so the 0–0 components are opposite in sign from the film spectra. However, the vibronic series shows no sign change but has a negative band at 400 nm, which is indicative of π–π stacking of anthracenes [9]. The lipidic LPL (turquoise) is dominated by the π–π coupled components showing little or no evidence of 254 nm/378 nm coupling. Instead, in the LPL and fluorescence, we see evidence of anthracene coupling to a trace impurity of tetracene, resulting in significant tetracene emission. Data are replotted from [10].

Fluorescence experiments usually involve an isotropic sample and unpolarised light. Signal intensity depends on both the population of emitting molecules and the probability of the transition occurring back to the ground state by emission of a photon, rather than by radiationless decay. For most fluorophores, fluorescence is the electric dipole transition probability from the first excited state to the ground state. So, the isotropic experimental intensity may be written as

(1) $$I_{iso-iso}=\kappa \left(\mu_{Y}^{fi}\mu_{Y}^{if}+\mu_{Z}^{fi}\mu_{Z}^{if}\right)=\frac{2\kappa \left(\mu_{x}^{fi}\mu_{x}^{if}+\mu_{y}^{fi}\mu_{y}^{if}+\mu_{z}^{fi}\mu_{z}^{if}\right)}{3}=\frac{2\kappa }{3}\mu^{fi}\cdot \mu^{if}$$

where κ is a constant that includes the probability of being in the (first) excited state f and, for example, $\mu_{Y}^{fi}$ is the *Y*-component of the electric dipole-allowed transition moment from the ground (*i*) to the excited state. The factor of 2/3 arises from isotropic orientational averaging, which relates the laboratory-frame components to the molecular frame components (Figure 1a). In this review, the axis systems of Figure 1a are used except for FPA. The total fluorescence (often measured as a direct current, DC) signal is collected without polarisation analysis, rendering it effectively invariant to molecular orientation and rotational dynamics.

When designing a fluorescence experiment, one can choose to fix the excitation wavelength and scan the emission wavelength (emission spectrum) or fix the emission wavelength and scan the excitation wavelength (excitation spectrum). Unless the molecule changes geometry after excitation, the excitation spectrum mimics the absorbance spectrum with each transition intensity scaled by its quantum yield. The lowest energy emission band is usually relatively enhanced compared with absorbance magnitudes as higher states tend to have more pathways to lose energy than the first excited state. Figure 1b includes the LD (which occurs at absorbance positions) and fluorescence of anthracene in different environments. Both absorbance and fluorescence have a transition between the ground vibrational levels of the ground and first electronic excited states, 0←0 transition at 378 nm. The absorbance then has components at shorter wavelengths (higher energies) for transitions from the ground vibrational level of the ground electronic state to higher vibrational levels of the first electronic excited state. Conversely, the fluorescence has components at longer wavelengths with transitions from the ground vibrational level of the first electronic excited state to higher vibrational levels of the ground state higher.

## Fluorescence polarisation anisotropy

**Figure 2:**
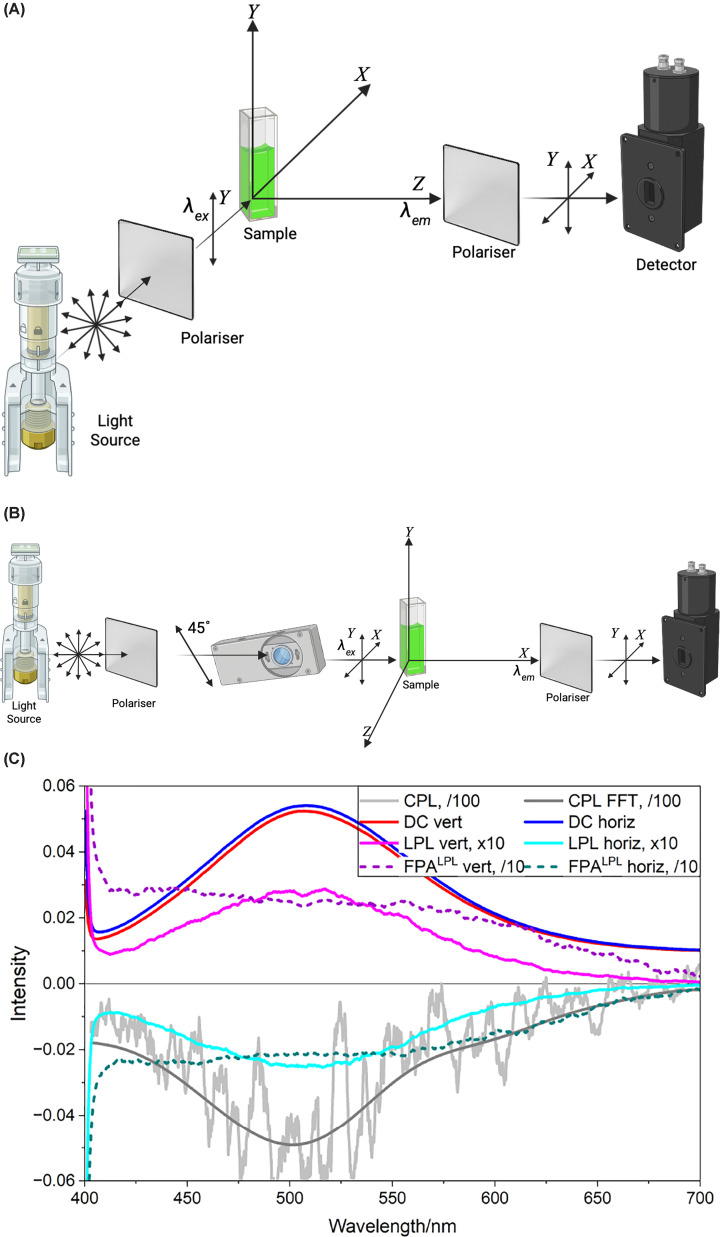
Axis systems used in FPA experiments, and example LPL/CPL spectra (**A**) Axis system of a standard FPA experiment using the axis system defined in Figure 1. (**B**) Axis system for measuring polarisation as an FPA alternative using an instrument configured for LPL. (**C**) Spectra of fluorophore DAPI (Figure 3c) bound to calf thymus DNA (50 μM:50 μM): fluorescence (DC), CPL (see below), and LPL (see below) spectra collected with a polariser inserted in the light beam with vertical (Y) or horizontal (Z) orientation. The ratios of the LPL and DC give the first wavelength-scanning *FPA^LPL^* measurements (collected for this review). The *FPA^LPL^* signals (dotted lines) are constant across a single isolated transition with the sign depending on the polariser orientation. FFT denotes fast Fourier transform option in Jasco Spectra Manager 2. Data collected on a Jasco CPL/LPL hybrid spectrometer [10].

When a fixed molecule is excited into its first excited state with light polarised along its electric dipole transition moment, then any fluorescence will also be along the same direction. If a molecule is free to rotate, its emission may be in another direction. FPA is usually defined as

(2) $$FPA=\frac{F_{YY}-GF_{YX}}{F_{YY}+GF_{YX}+GF_{YZ}}=\frac{F_{YY}-GF_{YX}}{F_{YY}+2GF_{YX}}$$

where *F_YX_* denotes the measured emission of photons polarised along the X-direction after excitation with Y-polarised photons, the denominator is the total emission, and *G* = *F_ZY_*/*F_ZX_* allows for the fact that the instrument may not transmit light with the same efficiency in the two directions (Figure 2a). FPA is therefore a measure of the retention of orientation during the time between excitation and emission. A molecule that does not rotate will have *F_YX_* = 0 and maximum FPA. A quickly rotating molecule has an FPA close to zero. The rotation of molecules in solution is largely dictated by Brownian motion, as noted by Weigert in 1920 [3]. Therefore, the retention of the polarisation of light between excitation and emission increases with increasing molecular size, viscosity of the medium, and decreasing temperature—all of which slow particle motion. Consequently, FPA is well suited to measuring the binding of a small molecule to a larger one, as a small fluorophore's FPA will significantly increase upon binding to a large molecule such as a protein or DNA.

FPA experiments are usually performed in a standard 90° fluorimeter (Figure 2a) with polarisers in both the excitation and emission beams whose orientation is changed to evaluate (Eqn(2)). The polariser orientations are usually expressed as vertical or horizontal, but for consistency with the techniques discussed in this article, a laboratory-fixed axis system {*X*, *Y*, *Z*} is used here. While some instruments measure FPA automatically, it is usually only at one wavelength. To measure a full frequency-resolved FPA spectrum, one needs either to collate multiple individual data points or to rotate polarisers and measure and subsequently combine two spectra if G = 1 or four spectra if G ≠ 1 (Eqn(2)).

Although Jameson and Ross [2] concluded that the first observation of FPA was in 1920, it appears that the first application for biomolecules was Laurence’s work 31 years later in 1951 to determine the binding of fluorescent ligands to bovine serum albumin [11]. They attribute the introduction of the term to Jabłoński [12]. Jameson and Ross also reviewed FPA applications to study proteases and kinases. Given how FPA removes background signals from non-fluorophores and small molecule fluorophores, it is disappointing how little it has been used. This is probably the consequence of the experiments being rather tedious.

The work of Jiang et al. illustrates how FPA can be used to provide information about biomolecule interactions. They used the fluorescence metal complex [Ru(bpy)_2_(dpqp)]^2+^ (bpy = 2,2′-bipyridine; dpqp = pyrazino[2′,3′:5,6]pyrazino[2,3-f][1,10]phenanthroline) to follow the kinetics of Alzheimer’s Aβ_1−42_ amyloid fibril formation. Although the precise methodology was ambiguous, the data are likely from multiple single-wavelength FPA measurements with excitation ranging from 350 to 490 nm and emission collected at 620 nm [13]. As the Aβ_1−42_ forms large soluble oligomers to which [Ru(bpy)_2_(dpqp)]^2+^ binds, the fluorophore rotational correlation time increases, and so does its FPA.

An alternate fluorescence anisotropy definition is to use the so-called polarisation [2] which has the simple sum of the polarised emissions as the denominator:

(3) $$FPA^{LPL}=\frac{F_{YY}-F_{YZ}}{F_{YY}+F_{YZ}}$$

With an instrument able to measure LPL, see below, which is configured to ensure G = 1, then by inserting a vertical Y-polariser (or Z polariser with inversion of Y and Z in (Eqn(3))) in the excitation beam, the LPL× 2ln(10) (see below) is the numerator of (Eqn(3)) and the DC is the denominator. Figure 2b illustrates this instrument configuration. As far as we are aware *FPA^LPL^* has not been measured directly in this way until the data presented in Figure 2c.

In addition to ligand binding, FPA has also been used to help assign transition polarisations by exciting into a higher excited state and measuring emission from the first excited state since, in this case, assuming no change in orientation,

(4) $$FPA=\frac{2}{5}\left(3{cos}^{2}\chi -1\right)$$

where χ is the angle between the excitation and emission transition dipole moments [14,15]. Alternatively, when *FPA^LPL^* is measured χ can be determined using

(5) $$FPA^{LPL}=\frac{3\cos^{2}\chi -1}{\cos^{2}\chi +3}$$

These can be used to choose between the two polarisation assignments identified in a linear dichroism experiment.

## Fluorescence-detected circular dichroism and fluorescence-detected linear dichroism

**Figure 3:**
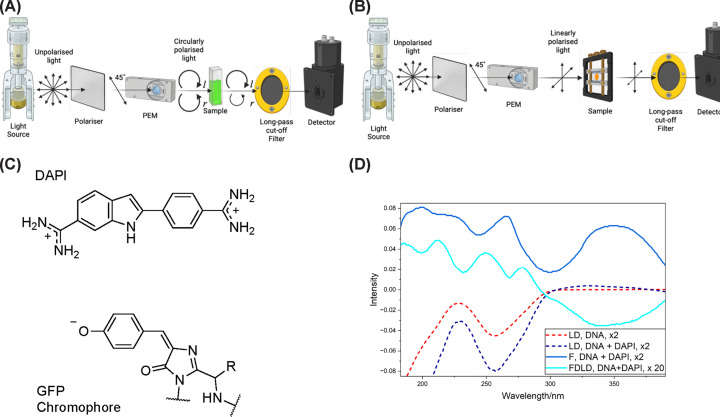
Schematic illustrations of FDCD/FDLD and fluorophores with associated FDLD spectra. (**A**) Schematic illustration of FDCD. (**B**) Schematic illustration of FDLD. (**C**) Some fluorophores used in this work. (**D**) Overlay of Couette flow-oriented LD of DNA and LD, fluorescence and FDLD of DAPI (30 μM) with DNA (500 μM base). Replotted from data collected on the AU-CD beam line of the ASTRID2 synchrotron light source at the Department of Physics and Astronomy, Aarhus University, Denmark [10].

Fluorescence-detected absorbance (FDA), CD, and LD involve scanning the excitation wavelength with alternating polarisations of light and collecting the photons emitted for each polarisation. The FDA spectrum uses unpolarised light (or the sums of left/right or horizontal/vertical) and is the standard fluorescence excitation spectrum, which can be collected in any fluorimeter. FDCD and FDLD require polarised light beams and 180° detection (or more complicated data analysis). While it is possible to rotate a polariser and collect independent spectra with a standard fluorimeter, artefacts often exceed the signal, so FDCD and FDLD are typically performed by inserting an appropriate long-pass filter in a CD/LD instrument between the sample and the detector to block the incident light while allowing emitted photons of longer wavelength to pass (Figure 3a,b). Johannson et al. (1981) reported what was likely the first FDLD measurement, using a dye laser, an integrating sphere to collect all fluorescence, and a long-pass filter in the emission beam, to record 12 points across 60 nm of an absorbance band [16]. Presumably, they rotated the sample rather than the laser, though the laser polarisation could have been set at 45° and a polariser rotated to achieve the same result.

Choosing the wavelength of long-pass filters often involves some sort of compromise, as the lowest-energy absorbance band overlaps with the highest-energy fluorescence band. Some CPL/LPL instruments can be configured to measure FD spectra using two monochromators rather than an excitation monochromator and emission filter. However, this requires the excitation light to be polarised, the emission detector to be locked into the excitation photoelastic modulator (PEM, which performs the polarisation), and the emission PEM to be off. In our hybrid J 1500/CPL 300 instrument, the PEMs encode CD at 50 kHz and LD at 100 kHz [17].

When the FDA, FDCD, and FDLD intensities are plotted as a function of excitation wavelength, they are proportional to the product of the fluorophore’s A, CD or LD and the quantum yield of each absorbing transition. FDCD and FDLD have the opposite sign from CD and LD because CD/LD measures transmitted light (what is not absorbed), whereas FD analogues track absorbed light:

(6) FDCD=-Φ×CD and FDLD = -Φ×LD

where Φ is the quantum yield of the transition of interest. For non-fluorescing compounds, Φ = 0. Unlike with absorbance spectroscopy, fluorescence-detected spectroscopies isolate the spectrum for the fluorophore, avoid most scattering artefacts, and often enhance the lowest energy bands (Figure 3b). The main disadvantages are the imperfect cut-off filters near the nominal wavelength and often low signal-to-noise. Using synchrotron radiation significantly improves FD experiments as they provide 3–6 orders of magnitude higher photon flux in the vacuum-UV region (160–200) compared with xenon lamps, dramatically improving signal to noise [10]. Figure 3d shows that the longest-wavelength FDLD band of 4′,6-diamidino-2-phenylindole (DAPI) binding to DNA is enhanced, and we can see multiple bands between 220 nm and 350 nm which are not apparent under the DNA LD in the LD spectrum.

## Circularly polarised luminescence

The instrumental setup for CPL (and LPL) operates in reverse from FDCD (and FDLD) with unpolarised light exciting the sample and a photoelastic modulator in the emission path enabling differential detection of the emitted polarisation states. Thus

(7) $$CPL=\left(F_{l}-F_{r}\right)$$

The ratio of the differential emission to the total emission is known as the dissymmetry factor. However, care must be taken when comparing published dissymmetry factors. On our Jasco J1500 CD/CPL instrument, using the axis system of Figure 1a, the CD and CPL dissymmetry factors in terms of the measured signals are [6,18]

(8) $$g^{CD}=\frac{CD}{32980\times Absorbance}=\frac{\left(A_{l}-A_{r}\right)}{32980\times \mathrm{Absorbance}}$$

(9) $$g^{CPL}=\frac{2\ln(10)CPL}{32980\times DC\;signal}=\frac{-2\ln(10)\left(F_{l}-F_{r}\right)}{32980\times \left(F_{l}+F_{r}\right)}$$

When interpreting reported values, spectroscopists should check for the factors 2ln(10) and 32 980 and whether or not their instrument software uses the sign convention in (Eqn(8) and Eqn(9)). On our instrument, *g^CD^* effectively carries a factor of 2, as the absorbance readout is averaged over the two polarisations. The factor 32 980 is required if the units plotted are mdeg. Most software sets the CPL sign to match that of the CD. For an isolated lowest energy absorbance band with no geometry change in the excited state before emission, *g^CD^* = *g^CPL^*.

CPL, like CD, is weak due to the dependence on magnetic dipole transition moments. High-power laser excitation light improves signal quality [19], but limits the fluorophores that can be studied. Linear and circular birefringence in the sample and/or optical path can interconvert polarisation states during propagation and contribute artefacts to measured spectra [20]. However, 180° detection geometry and depolarised excitation minimise such artefacts. Oriented samples can contribute significant linear contributions to CD and CPL. This is usually very clear if the samples are rotated. Paired measurement of both CD and CPL on the same sample in the same instrument is useful, but not possible with most commercial instruments. Accordingly, CPL and even more so LPL (see below) are not commonly used largely due to unavailability of appropriate instrumentation. Nonetheless, the potential of the techniques is illustrated with some examples.

## Intrinsic protein CPL

**Figure 4:**
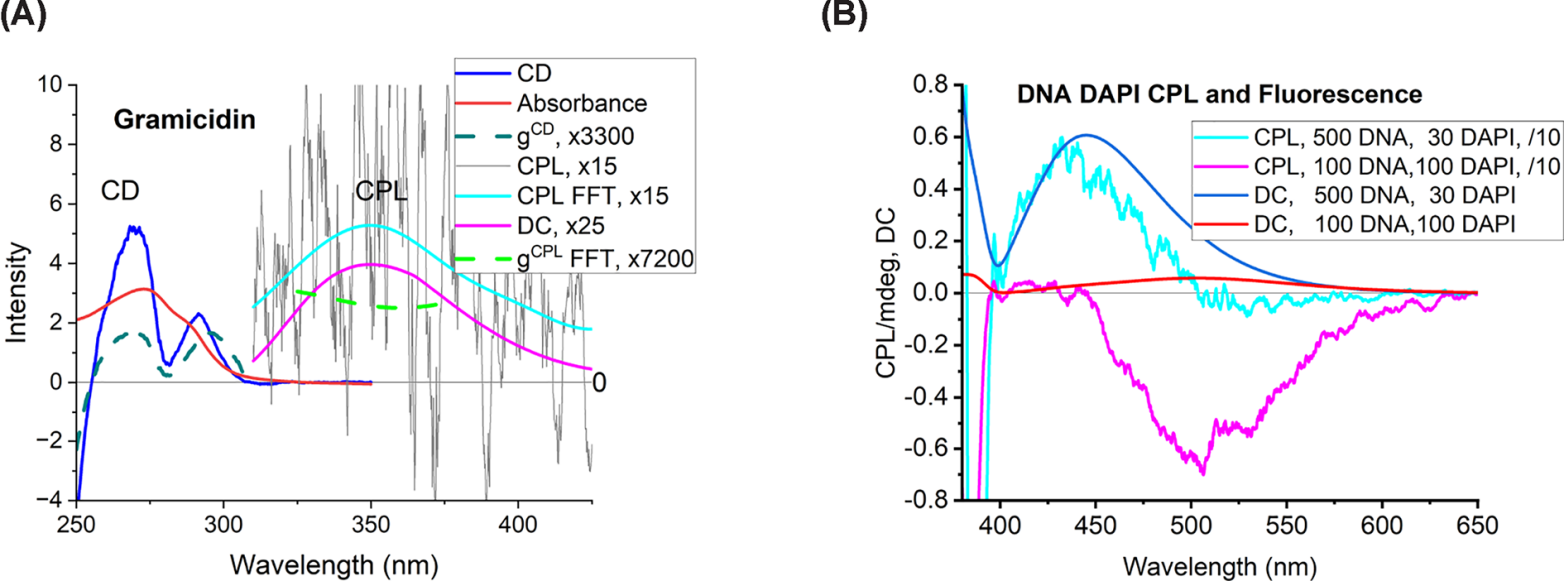
Example CD/CPL spectra (**A**) CD, absorbance, gCD, CPL (35 nm excitation bandwidth, 30 nm emission bandwidth, 4 s response time, 22 scans), fluorescence (DC), and gCPL of gramicidin (3 mg/ml in methanol). FFT denotes fast Fourier transform performed by the Jasco software. FFT denotes fast Fourier transform option in Jasco Spectra Manager 2. (**B**) CPL of calf thymus DNA with bound fluorophore DAPI at the ratios (in μM) indicated in the legend showing how the spectrum changes with binding mode [10].

Intrinsic protein fluorescence has a wide range of applications, yet few examples of protein CPL exist. Gussakovsky et al. [21,22], following Schlessinger et al. [23,24], reported CPL to probe protein structure, function, and folding. They reported only the *g^CPL^* and sometimes the fluorescence spectrum. They stated a 10% error on the *g^CPL^*, suggesting either more favourable samples or better instrumentation than ours and other reports. Our CPL measurements from the three tryptophans in the fifteen-residue peptide gramicidin show a noisy positive signal; the dissymmetry factor is 4.5 × 10^−5^, which is similar to the long wavelength band of the CD spectrum (Figure 4a).

Green fluorescence protein (GFP) has a strong intrinsic fluorophore (Figure 3c) buried in a β-barrel, plus a tryptophan and multiple tyrosines. Upon excitation at ∼395 nm, the neutral fluorophore loses a proton and emits light at 510 nm from the anionic deprotonated form [25]. Published GFP CD spectra often have signals at 400 nm and 480 nm but in varying amounts. Goto et al. [26] measured a positive 540 nm CPL for GFP. Their CD was negative above 430 nm (the anionic form), which is consistent with their definition of CPL being opposite of (Eqn(8)). They reported *g^CPL^* ≈ 0.002, about 50 times greater than that of gramicidin. Their long wavelength *g^CD^* is an order of magnitude smaller, consistent with excited state deprotonation enhancing emission.

## Extrinsic protein CPL

As summarised by Dai et al. [27], most protein CPL reports involve efficient fluorophores bound to peptide or protein structures by coordination, covalent synthesis, or noncovalent interactions. The coordination is mainly lanthanide complexes which bind to biomolecules (discussed below). Rybicka et al. [28] measured the CPL that resulted from the achiral thioflavin T (3,6-dimethyl-2-(4-dimethylaminophenyl)-benzo-thiazolium cation, ThT) bound noncovalently to two forms of insulin amyloid fibres. The two forms gave positive or negative ThT long wavelength CD signals and matching short wavelength CPL signals (transitions between ground state and first excited state), while gCPL was 40% more than gCD because free ThT contributes to the total absorbance (the gCD denominator) but not to the fluorescence (gCPL denominator). Chiroptical inversion of amyloid fibrils may have biological significance, which motivated Li et al. [29] to explore ThT CPL inversion. In a more complex example, Deng et al. created helical fibres self-assembled from N-(9-fluorenylmethoxycarbonyl)-protected glutamic acid and purine nucleobases which bound ThT and produced a CPL signal, but they had no success with pyrimidines [30].

## Nucleic acid dye CPL

Probably the first use of CPL to study DNA-associated fluorophores was by Górecki et al. in 2017 [31]. They detected it at 90° from the incident light and they noted artefacts e.g., the achiral fluorophore DAPI gave a CPL signal. Using a 180° configuration, we later showed that CPL reveals DNA binding mode changes for DAPI with different stoichiometry (Figure 4b) [10]. Jiang et al. showed that AT-rich double-stranded DNA induces CPL into achiral carbazole-based biscyanine fluorophores that bind into the minor groove of DNA [32]. Right-handed and left-handed duplex DNA gave positive and negative CPL signals, respectively. By adjusting pH to form other DNA structures, such as triplex with a third strand in the DNA minor groove, the CPL switched off. They did not report the CD signals, but their CPL signs match previous low-loading-induced CD for long-axis transitions of minor groove dyes, e.g., DAPI [10,33] and Hoechst 33258 [14] with right-handed DNA. Chen et al. showed that changes in intensity, sign and wavelength of the CPL of ThT can be used to follow the formation of parallel and antiparallel and left- and right-handed DNA G-quartet structures [32]. There are yet to be any reports using the CPL of nucleic acid dyes to study RNA systems.

## CPL of amino acid–lanthanide and protein–lanthanide systems

**Figure 5:**
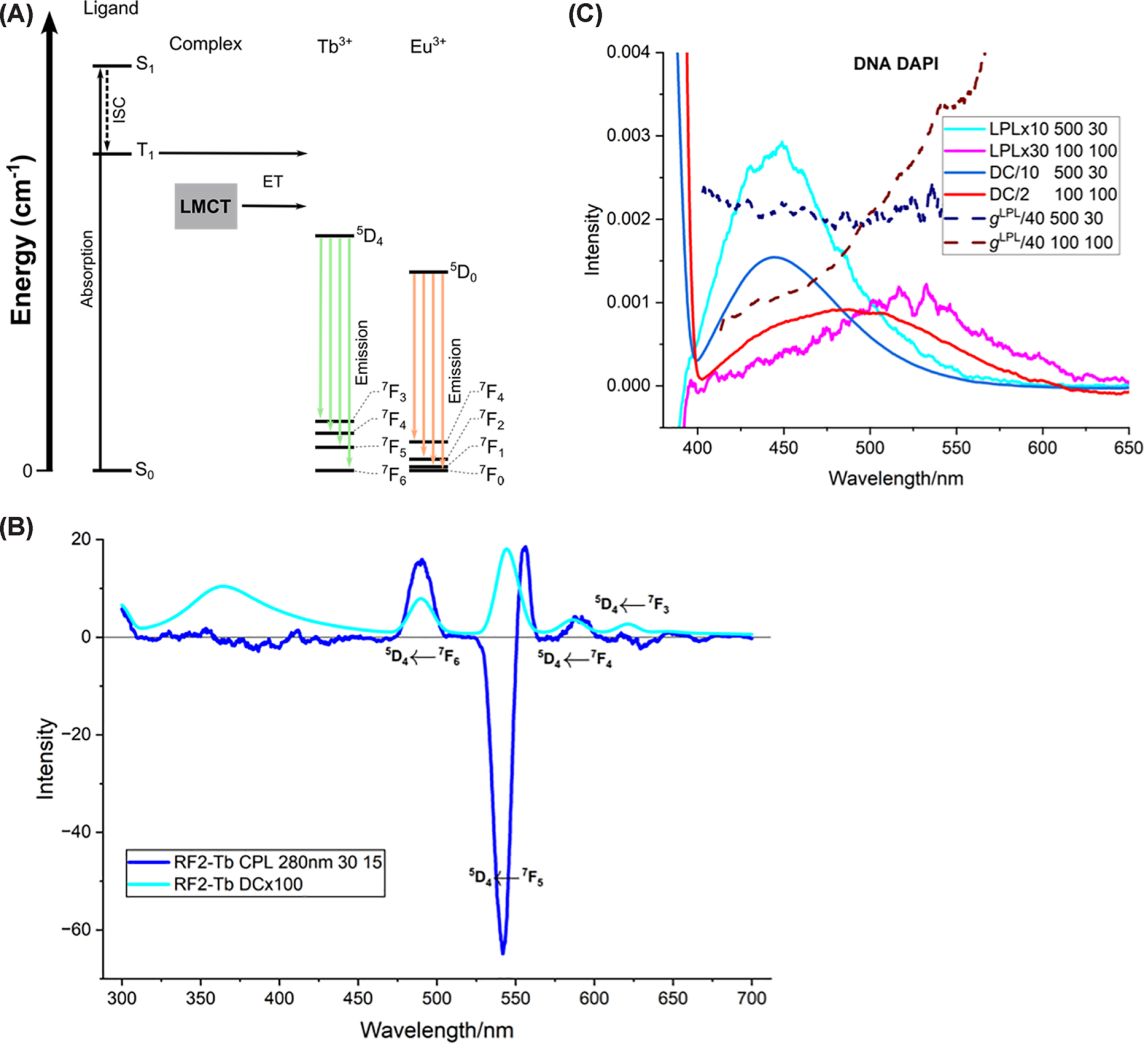
CPL of Tb^3+^ and LPL of DAPI (**A**) Jabłoński diagram for lanthanide emission. For antenna-mediated sensitisation, a ligand is excited to S1, which undergoes intersystem crossing (ISC) to T1, followed by energy transfer (ET) to the Ln^3+^. A high-lying ligand-to-metal charge-transfer (LMCT) band is shown as an alternative excitation route. Once energy is transferred into a magnetic dipole allowed state, emission is then observed for f→f transitions. For Tb^3+^, the commonly observed bands are ^5^D_4_→^7^F_6_ ≈ 488–495 nm, ^5^D_4_→^7^F_5_ ≈ 541–547 nm, ^5^D_4_→^7^F_4_ ≈ 583–587 nm, and ^5^D_4_→^7^F_3_ ≈ 619–624 nm. For Eu^3+^, the commonly observed bands are ^5^D_0_→^7^F_0_ ≈ 580 nm, ^5^D_0_→^7^F_1_ ≈ 593 nm (magnetic-dipole, least environment-dependent), ^5^D_0_→^7^F_2_ ≈ 611–617 nm (hypersensitive), ^5^D_0_→^7^F_3_ ≈ 645–653 nm, and ^5^D_0_→^7^F_4_ ≈ 685–705 nm. The notation used for Ln states is manoj, where S is the total spin, *L* is the total orbital angular momentum (with letters S denoting L = 0, P L = 1, increasing in order D, F, G, H, I, etc.), and J is the total angular momentum quantum number. Magnetic dipole allowed transitions involve Δ*J* = 0, ±1 (except 0 to 0) and Δ*M_J_* = 0, ±1 (also Δ*S* = 0 and Δ*L* = 1). (**B**) CPL and fluorescence RF2 (1 mM) with equimolar Tb^3+^ at pH 7.0. (**C**) LPL and fluorescence of DNA/DAPI systems at low (500 μM DNA/30 μM DAPI) and high (100 μM DNA/10 μM DAPI) dye loading.

Much of the published CPL data concerns lanthanides (Ln^3+^) because their emission states are electric dipole forbidden from the ground state but many are magnetic dipole allowed, yielding larger dissymmetry factors. Intrinsic Ln^3+^ absorbance is weak and experiments usually excite an ‘antenna’ ligand band that transfers energy into a magnetic dipole-allowed state. Luminescence is then observed for f→f transitions (Figure 5a). In a beautiful paper published in 1977, Brittain and Richardson [34] reported Ln-amino acid CPL with europium (Eu^3+^) and terbium (Tb^3+^) complexes. They cited only four papers in total and presented data for Tb^3+^ and Eu^3+^ bound to amino acids L-aspartic acid (L-asp), L-serine (L-ser), L-threonine (L-thr), and L-histidine (L-his) in D2O as a function of pH. Their aim was to use lanthanides as calcium replacements to probe Ca^2+^ binding in proteins. Excitation at 365 nm for (Tb^3+^) and 310 nm for (Eu^3+^), which does not match f→f transitions, nor in-ligand transitions, but given the electron-donating nature of amino acids, it is consistent with ligand-to-metal charge-transfer (LMCT) transitions. They advocate CPL over CD for their work because lanthanide emission is strong, whereas their absorbance is small. Changes in pH tuned the nature of the complex, with variable numbers of distinct bands observed. For the Tb^3+^/L-asp, ^5^D_4_→^7^F_5_ transition, 19 of the 99 components are magnetic dipole allowed. At pH 9, they resolved six different components (a negative dip is probably a positive signal overlaid by larger negative ones). Spalding and Brittain in 1985 [31] enhanced the terbium luminescence by complexing it to ethylenediaminetetraacetic acid (EDTA), adding amino acids and raising the pH to 8–10.

In 1981 De Jersey et al. observed enhanced 545 nm Tb^3+^ emission upon titrating aqueous TbCl_3_ with proteins near pH 6.6 while exciting tryptophan, ionised and unionised tyrosine, and phenylalanine, indicating the amino acids were sufficiently close to act as an antenna [35]. Çoruh and Riehl (1992) measured CPL of Tb^3+^ bound to trypsin, calmodulin and parvalbumin. Trypsin showed a distinct lineshape at the ^5^D_4_→^7^F_5_ transition, whereas the EF-hand proteins calmodulin and parvalbumin shared a bisignate couplet, suggesting similar coordination environments [36]. In Figure 4b, we present spectra of RF2, a *de novo* EF-hand protein [37], which shows the same bisignate signature at the ^5^D_4_→^7^F_5_, ^5^D_4_→^7^F_6_ and ^5^D_4_→^7^F_4_ transitions. We calculated the dissymmetry factors gCPL = +0.014 (491 nm, ^5^D_4_→^7^F_6_), −0.033 (536 nm, ^5^D_4_→^7^F_5_), +0.029 (558 nm, ^5^D_4_→^7^F_5_), and +0.012 (596 nm, ^5^D_4_→^7^F_4_). Amino acid lanthanide complexes can also enhance the CPL of other molecules (e.g. [38]) by exciting into Ln^3+^ LMCT bands which gain a chiral environment. Okutani et al. using the intensely luminescent achiral complexes [Eu(pda)_2_]^-^ (pda = 1,10-phenanthroline-2,9-dicarboxylic acid) shows red (570–700 nm) ^5^D_0_→^7^F_0_-4 transitions in the presence of arginine (pH 6) and histidine (pH 3), providing chiral sensing of amino acids. Other amino acids gave much weaker signals [39]. They attributed the CPL to chirality induced into the pda.

Krupová et al. used a Raman optical activity (ROA) instrument as described below to measure the CPL induced into EuCl_3_ and Na_3_[Eu(DPA)_3_] (where DPA = pyridine-2,6-dicarboxylic acid) between 580 nm and 600 nm in the presence of glutamic acid enantiomers and lysozyme fibrils. They found that protein and metal complex charges affected whether a complex was formed and CPL observed.

## CPL of lanthanides and other biomolecules

Shuvaev et al. examined CPL of an achiral bimetallic [EuL]Zn^2+^ (L = picolylamine) in the presence of ADP and ATP, and found opposite handedness in the ^5^D_0_→^7^F_4_ Eu band with 335 nm excitation and detection from 570 nm upwards [40]. The nucleotide binds through a phosphate that displaces a Eu-bound water bridge to Zn.

Wu, Bouř and Andrushchenko [41] used an ROA instrument equipped with a 532 nm laser and a standard 538–610 nm ROA detection range to measure beautiful CPL spectra of direct excitation of the lanthanide in dGMP-Eu^3+^ and DNA-Eu^3+^ complexes, where they observed dependencies on pH, DNA structure, and binding stoichiometry. They mainly probed the ^5^D_0_→^7^F_1_ (1800–2000 cm^−1^ ≈ 588–595 nm) transition but also in one case saw weak CPL for ^5^D_0_→^7^F_0_ (1530 cm^−1^ ≈ 536 nm). Nakamura et al. illustrated how a europium metal complex could hybridise to DNA in surfactant films, giving high fluorescence and CPL. Their focus was on how DNA enhanced the lanthanide fluorescence rather than using it to study the DNA.

As a final example, we note that Wu et al. used the above ROA method to get very different signatures for different sugars in the presence of aqueous EuCl_3_, NaEu(EDTA), and Na_2_Eu(diethylenetriaminepentaacetic acid). The authors were unsure how the lanthanide complexes interacted with the sugars.

## Linearly polarised luminescence

Analogous to CPL, LPL excites with unpolarised light and measures the difference between horizontally and vertically polarised emission. The LD and LPL dissymmetry factors are for any given molecule (using the axis system of Figure 1a) [6,18]:

(10) $$g^{LD}=\frac{LD}{Absorbance}=\frac{A_{Z}-A_{Y}}{\left(A_{Z}+A_{Y}\right)/2}=3/2S(\left\langle 3\cos^{2}\alpha \right\rangle -1)$$

and

(11) $$g^{LPL}=2\ln\left(10\right)\frac{LPL}{DC\;signal}=2\ln\left(10\right)\frac{F_{∥}-F_{⊥}}{\left(F_{∥}+F_{⊥}\right)}=-2\ln(10)\frac{F_{Z}-F_{Y}}{\left(F_{Y}+F_{Z}\right)}$$

The orientation parameter *S* reflects the degree of alignment achieved under the applied shear field, and α is the angle the transition moment makes with the orientation direction [7,8]. The LPL of a molecule also depends on its orientation parameter. As with CPL, spectroscopists should check whether the 2*ln*(10) is included and which sign convention the software uses. Our software currently plots LPL of opposite sign from the LD as it was chosen to match FDLD. For publication, we invert the LPL from the experiment so that *g^LD^* = *g^LPL^* for an isolated lowest energy absorbance band with no geometry change in the excited state before emission.

The signal-to-noise ratio of LPL is much better than CPL, but sample orientation is required. On its simplest level, LPL mirrors LD (with the mirror at the 378 nm 0–0 transition) as illustrated in Figure 1b (pink and purple) for anthracene in a stretched polyethylene film and a flow-oriented liposome (blue and turquoise). Figure 5c clearly shows the change in binding mode of 4′,6-diamidino-2-phenylindole (DAPI) with loading on DNA. As with CPL, birefringence artefacts can be minimised through the 180° detection and the use of depolarised incident light.

## Summary and conclusions

Polarised luminescence spectroscopies provide additional dimensions to fluorescence measurements that reveal biomolecular structure, orientation, chirality, and dynamics. FPA monitors rotational mobility, distinguishing bound from unbound fluorophores without interference from non-emitting species. FDCD and FDLD isolate fluorophore absorption spectra from backgrounds, revealing structural details obscured in standard absorbance measurements. CPL probes fluorophore environment chirality and can provide large dissymmetry factors for lanthanide-luminescent systems, which would be valuable for characterising binding environments. LPL provides relative orientation information with superior signal-to-noise compared with CPL, though requiring sample alignment. Although in this article we do not address how polarised spectroscopies could enhance exciton coupling or Förster resonance energy transfer or quenching experiments, we anticipate they would add an additional dimension to the data acquired and so help support analysis of, e.g., multiple tryptophans in a protein.

Because LPL requires oriented fluorophores, either through sample alignment or through binding to chiral/oriented biomolecular environments, it contains information about the local three-dimensional geometry that is absent from isotropic luminescence measurements. However, the field still lacks the simple interpretive heuristics that have made CD spectroscopy, for example, widely accessible. Systematic studies establishing when each technique provides unique structural information would accelerate adoption beyond specialist laboratories. For biomolecular systems, the greatest opportunities lie in exploiting the background-free nature of these techniques to study binding and folding in complex mixtures where other methods fail. Wider adoption will also require improved access to instrumentation, particularly for wavelength scanning CPL and LPL measurements, currently available only on few commercial instruments.

## Perspectives

- Polarised luminescence spectroscopies reveal biomolecular interactions invisible to isotropic fluorescence and eliminate interference from non-emitting backgrounds that plague conventional methods. These techniques enable quantitative analysis of binding modes, orientations, and proportions in complex biological mixtures.
- FPA monitors binding events through changes in complex mobility, while fluorescence-detected circular and linear dichroism isolate fluorophore absorption spectra from complex backgrounds. CPL provides sensitive measurements of chiral environments, while LPL directly reports on molecular orientation.
- The future of polarised luminescence spectroscopy depends on improved instrumentation and the development of robust interpretative frameworks. Wider adoption will require commercial instruments capable of wavelength-scanning CPL and LPL measurements, and systematic studies to establish interpretive principles for polarised luminescence spectra.

## Data Availability

Data in this review are drawn from literature cited in the article. Original data underlying Figure 5b are available on Zenodo at DOI: 10.5281/zenodo.17410649.

## Open Access

Open access for this article was enabled by the participation of The Australian National University in an all-inclusive Read & Publish agreement with Portland Press and the Biochemical Society under an agreement with CAUL.

## Abbreviations

CD: circular dichroism
CPL: circularly polarised luminescence
DAPI: 4′6-diamidino-2-phenylindole
EDTA: ethylenediaminetetraacetic acid
FDA: fluorescence detected absorbance
FDCD: fluorescence detected circular dichroism
FDLD: fluorescence detected linear dichroism
FPA: fluorescence polarisation anisotropy
GFP: green fluorescence protein
LD: linear dichroism
LMCT: ligand-to-metal charge-transfer
LPL: linearly polarised luminescence
ROA: Raman optical activity
ThT: thioflavin T
